# Midterm Outcomes With the Nellix Endograft Alone or With Chimneys

**DOI:** 10.1016/j.ejvsvf.2024.06.001

**Published:** 2024-06-22

**Authors:** Salomé Kuntz, Céline Deslarzes, Alexandre Than Vinh Nguyen, Alban Longchamp, Rosalinda D'Amico, Justine Longchamp, Anne Lejay, Nabil Chakfé, Sébastien Déglise

**Affiliations:** a Department of Vascular Surgery and Kidney Transplantation, University of Strasbourg, France; b Gepromed, Strasbourg, France; c Department of Vascular Surgery, Lausanne University Hospital, Lausanne, Switzerland

**Keywords:** Abdominal aortic aneurysm, Endoleak, Endovascular Aneurysm Sealing, EVAS, Nellix

## Abstract

**Introduction:**

Endovascular aneurysm sealing (EVAS) appeared to be an innovative alternative to conventional endovascular abdominal aortic aneurysm repair. However, high rates of midterm failure of EVAS led to withdrawal of the device from the market. The study aim was to report midterm outcomes of patients treated with EVAS alone or associated with chimneys (Ch-EVAS) and the management of their complications.

**Methods:**

In this single centre study, all consecutive Nellix implants between 2013 and 2016 were included. The primary endpoint was device failure: (1) a triad of caudal migration of the Nellix stents >5 mm, separation of the endobags (>5 mm), and sac enlargement (>5 mm), with or without visible endoleak, (2) secondary aneurysm rupture, (3) surgical explant of the graft, or (4) any intervention for a type I endoleak. Overall mortality, aneurysm related mortality, and re-intervention rates were analysed.

**Results:**

Fifty patients (male *n* = 43, female *n* = 7) were included. Median follow-up was 3.05 years (interquartile range [IQR] 0.52, 4.63) and follow up index was 0.51 (IQR 0.10, 0.88). Device failures occurred in 17 patients (34%). Overall and aneurysm related mortality rates during the follow up period were 30% and 13%. Fourteen (28%) patients required re-interventions. Five EVAS patients (17%) presented with complications. Type Ia endoleaks were managed by device explantation for three patients, and endovascular aneurysm repair in Nellix for two patients. Type Ib endoleaks were managed with an iliac branched device and limb extension. Nine Ch-EVAS patients (42.9%) presented with complications. Type Ia endoleaks were was managed by Nellix stent prolongation and renal extension, two multibranched thoraco-abdominal devices, and two device explantations. Type Ib endoleaks were managed by limb extension and stent complications by stent angioplasty and iliorenal bypass.

**Conclusion:**

The midterm outcome of EVAS is poor. All patients who underwent EVAS implantation must be informed and should undergo frequent surveillance. Open repair and device explantation should be considered as the primary treatment.

## Highlights

• Patients with Nellix EVAS system need to be part of an enhanced surveillance program.
• Mid-term follow up revealed a high incidence of failure leading to reinterventions.
• Median time of graft failure and reinterventions occurred around 3 years.
• Endovascular procedures should be considered for patients unfit for open explantation.
• Explanted Nellix need to be sent to GEPROMED for further biomechanical analysis.

## Introduction

Endovascular aneurysm repair (EVAR) has revolutionised the treatment of abdominal aortic aneurysms (AAAs) and accounts for 70% of elective repairs. However, open surgical repair remains the gold standard of care for patients considered fit enough to withstand major surgery and EVAR has been used increasingly in patients judged unfit.^1^ Despite its popularity and results of trials showing that the 30 day mortality rate in such patients is less than 2%,^2^ long term mortality and re-interventions seem to be higher than open repair.^3^ The technique is considered less invasive than conventional surgery, with the goal of eliminating the risk of rupture of the aneurysm by excluding the aneurysmal wall from systemic arterial pressure. However, incomplete sealing and endoleaks allow further expansion of the aneurysm with the potential for eventual rupture.^4^, ^5^, ^6^

Since the introduction of EVAR, many devices have been developed trying to solve the endoleak issue. Initial experience demonstrated the importance of device stability and studies showed that degenerative phenomena occurred in endoprostheses (nitinol and corrosion but also textile structure).^7^^,^^8^

Since 2013, the endovascular aneurysm sealing (EVAS) system with the Nellix endoprosthesis (Endologix, Inc., Irvine, CA, USA) has been developed to solve endoleak and stent graft migration. This new concept of aneurysm exclusion was based on polymer filled polyurethane bags surrounding balloon expandable stents covered with PTFE to completely seal the aortic aneurysm sac.

Enthusiasm was generated by very good technical success rates with low peri-operative complications and good short term one year outcomes.^9^^,^^10^ Using this device, the neck anatomy seemed to play a smaller role in aneurysm exclusion, allowing treatment of juxtarenal AAA (reducing the risk of gutters and using chimney grafting in combination with EVAS [Ch-EVAS], but debatable at the time), and procedure time was reduced (including radiation exposure).^11^^,^^12^

The goal of this new concept was to reduce the rate of type II endoleaks.^13^ The initial enthusiasm for this technique was significant. However, the EVAS investigational device exemption trial reported higher incidences of type Ia endoleak, graft migration, and secondary AAA rupture at two years,^14^ which resulted in refinement of the instructions for use. Significant midterm failures, at a median follow up of less than five years, were further demonstrated in larger series.^15^, ^16^, ^17^ Subsequently, EVAS was temporarily withdrawn from the market. However, since there are still many patients being treated with EVAS, it is mandatory to follow them and treat them in case of complications.^18^

The aim of this study was to report the management of midterm failure associated with the EVAS Nellix system.

## Materials and methods

### Design

**Figure 1:**
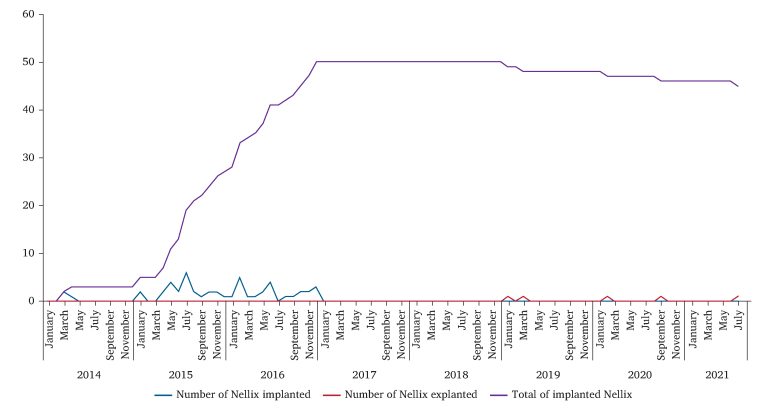
Implanted and explanted endovascular aneurysm sealing over time.

This was a single centre study of all consecutive patients treated with EVAS or Ch-EVAS treated in the Department of Vascular Surgery, University Hospital of Lausanne (Centre Hospitalier Universitaire Vaudois (Lausanne, Switzerland) (CHUV)) from 14 March 2014 to 31 December 2016. Surveillance was carried out until 15 August 2021 (Fig. 1). Patients’ data were collected retrospectively using applications of the CHUV, such as Soarian, Archimède, and Pacsweb, as well as external consultation reports in paper form. AAA morphology was analysed from abdominal computed tomography angiograms (CTA) performed pre-operatively, using the 3mensio application vascular 8.0 (Pie Medical Imaging, Bilthoven, The Netherlands).

### Population

Pre-operative work up included pre-assessment clinic attendance with baseline investigations for elective cases. A standardised post-operative surveillance protocol included CTA within three months of implantation, followed by combined duplex ultrasonography and CTA if needed.

The primary endpoint was device failure: (1) a triad of caudal migration of the Nellix stents >5 mm, separation of the endobags (>5 mm), and sac enlargement (>5 mm), with or without visible endoleak, (2) secondary aneurysm rupture, (3) surgical explant of the graft, or (4) any intervention for a type I endoleak.^19^ Re-intervention was defined as any procedure required to exclude the aortic aneurysm.

Follow up was calculated using total person years. Follow up index (FUI) was used to describe completeness of follow up.^20^ The overall mortality, aneurysm related mortality, and re-intervention rates were also analysed. Aneurysm related death was defined as death within 30 days of the index procedure, within 30 days of re-intervention, or secondary to AAA rupture. The primary objective of this study was to investigate device failure and its management, re-interventions, and subsequent outcome and mortality rate.

### Statistical analysis

Statistical analysis was executed on GraphPad Prism 8 (GraphPad Software, CA, USA).

## Results

A total of 50 (male *n* = 43, female *n* = 7) patients fulfilled the inclusion criteria and were analysed from the 144 EVAR procedures performed in the centre during the same time. The mean age ±standard deviation (SD) was 77.0 ± 7.0 years. Median follow up was 3.05 years (interquartile range [IQR] 0.52, 4.63) and the follow up index was 0.51 (IQR 0.10, 0.88). Nine patients (18%) were treated within the instructions for use (IFU). At three years, 16 patients were lost to follow up. Patient characteristics are given in Table 1. Twenty nine patients were treated with EVAS and 21 patients with Ch-EVAS. Indications for EVAS were infrarenal AAA (*n* = 25), and for elective repair of failing EVAR or open repair (*n* = 4). Indications for Ch-EVAS were AAA with iliac aneurysm and short neck (*n* = 1), elective repair of failing EVAR device or open repair (*n* = 3), Ch-EVAS for juxtarenal AAA (*n* = 16), and ruptured AAA (*n* = 1) (Table 2).

**Table 1:** Demographics of patients and characteristics of abdominal aortic aneurysms treated by Nellix.

Characteristics	Patients (*n* = 50, 100%)
*Patient characteristics*	
Age – y	77 ± 7
Male	43 (86)
*Comorbidity*	
Ischaemic heart disease	28 (56)
Hypertension	40 (80)
Heart failure	32 (64)
Chronic obstructive pulmonary disease	18 (36)
Stroke	4 (8)
Diabetes	8 (16)
Chronic kidney disease	17 (34)
Smoking	
Current	20 (40)
Ex-smoker	20 (40)

Data are presented as *n* (%).

**Table 2:** Indications for treatment of abdominal aortic aneurysms by Nellix.

Indication	Patients (*n* = 50)	Max aneurysm diameter – mm
EVAS infrarenal AAA	25	61.7 ± 10.4
EVAS for elective repair of failing EVAR/open repair	4	61.2 ± 10.3
Ch-EVAS infrarenal AAA + iliac and short neck	1	52
Ch-EVAS for juxtarenal AAA	16	62.4 ± 10.4
Ch-EVAS for elective repair of failing EVAR and or open repair	3	61.8 ± 11.4
Ch-EVAS for ruptured AAA	1	85

Data are presented as *n* and mean ± standard deviation EVAS = endovascular aneurysm sealing; AAA = abdominal aortic aneurysm; EVAR = endovascular aneurysm repair; Ch-EVAS = chimney grafting in combination with endovascular aneurysm sealing.

There were 18 (36 %) device failures. Thirteen (26%) of them had sac expansion associated with caudal migration of the EVAS Nellix stent and five (10%) presented with secondary AAA ruptures, and one patient presented with two AAA ruptures. Type I endoleaks were observed in 18 cases (type Ia *n* = 11, type Ib *n* = 7). Type II endoleaks were observed in eight cases. Fourteen (28%) patients required at least one re-intervention, five patients with EVAS and nine with Ch-EVAS. Six ruptures occurred and the median time to rupture was 55 months (IQR 51.5, 62.5). The median time (IQR) for graft failure was 47 months (32, 57) and median time to first re-intervention was 46 months (17, 53). The median time to re-interventions for EVAS alone was 54 months (IQR 45.6, 56.5) and for Ch-EVAS 38 months (IQR 6, 63). Re-interventions included two EVAR in Nellix procedures, two underwent branched thoraco-abdominal devices, five underwent device explantation, six patients underwent limb extension, two required embolisation, and three had stent angioplasty. Indications and treatments are summarised in Table 3 and Table 4. Overall, the 30 day mortality rate for all comers was 0%, overall mortality rate during the follow up period was 30%, and aneurysm related mortality rate during follow up was 13 %.

**Table 3:** Endovascular aneurysm sealing re-interventions.

Patient	Indication	Treatment	Time after first procedure – mo
1	EL 1a	Device explantation + aortobi-iliac bypass	58
2	EL 1a	Nellix in Nellix + chimney + sac embolisation	27
3	EL 1b – rupture	Iliac branched device + limb extension	55
	EL 1a – rupture	Device explantation + aorta to external iliac bypass + aorta to common iliac bypass	62
4	EL 1a	Device explantation – aortobi-iliac bypass	54
5	EL 1a – rupture	Endovascular aneurysm repair in Nellix	52

EL = Endoleak.

**Table 4:** Chimney grafting in combination with endovascular aneurysm sealing re-interventions.

Patient	Indication	Treatment	Time after first procedure – mo
1	EL 1a	Main Nellix stent prolongation + renal stent extension	6
	Stent occlusion	Renal angioplasty	21
	EL 1a	Multibranched thoraco-abdominal device	40
2	EL 1a + 1b	Thoracic endoprosthesis + visceral stents extension + limb extensions	46
3	EL 1b	Limb extension	38
4	EL 1b – Rupture	Bilateral limb extension	63
5	Stent occlusion	Failed renal angioplasty – iliorenal bypass	5
	EL 1a – rupture	Device explant – aortobifemora bypass	62
6	EL 1b + three	Visceral stent extension	7
	Stent occlusion	Failed renal angioplasty	14
	EL 1b	Limb extension	26
7	EL 1a +2	Embolisation sac + lombal arteries	6
	EL 2	Embolisation IMA	7
	EL 1a	Device explant – aortobi-iliac bypass	33
8	EL 1b – rupture	Limb extension	63
9	EL 1a	Multibranched thoraco-abdominal device + aorto-uni-iliac + femoro-femoral bypass + left limb embolisation	67

EL = Endoleak; IMA = inferior mesenteric artery.

### Devices failures management–re-interventions

**Figure 2:**
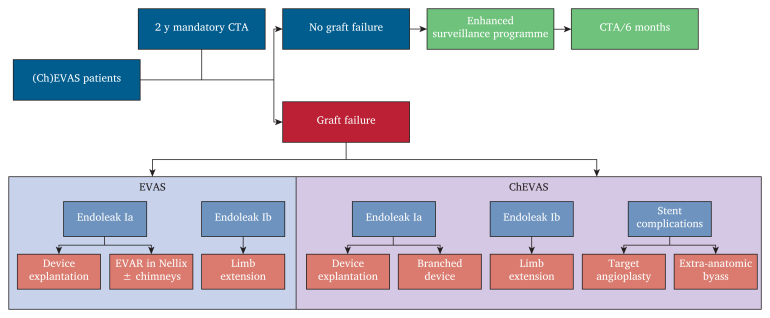
Management strategy for patients treated with endovascular aneurysm sealing (EVAS) and chimney grafting in combination with endovascular aneurysm sealing (ChEVAS). CTA = computed tomography angiography.

The management strategy is summarised in Fig. 2.

#### Managing endovascular aneurysm sealing complications

Five EVAS patients (17.2%) presented with complications. EVAS complications are listed in Table 3. All of them presented with type Ia endoleaks and one patient also presented with a rupture due to type Ib endoleak. Type Ia endoleak was managed by device explantation for three patients, and EVAR in Nellix for two patients. Type Ib endoleak was managed with an iliac branched device and limb extension.

Three patients underwent Nellix explantation and open surgical repair. An intraperitoneal approach was used in all cases with suprarenal cross clamping. One patient underwent Nellix explantation with suprarenal cross clamping, and an aortobi-iliac bypass 56 months after the first operation. One patient was treated with an aorta to left external iliac artery and aorta to right common iliac artery bypass, after a first procedure of limb extension. One patient presented bilateral type Ib endoleaks 54 months after the first procedure. He was treated with an emergency endovascular procedure with bilateral limb extension and unilateral iliac branched device. He presented again, 62 months after the first procedure, with a dislocation of the left Nellix stent with misplacement of the endobag and sac enlargement. He underwent Nellix explantation with an aorta to left external iliac artery and right aorta to common iliac artery bypass. One patient showed caudal migration of the right stent, separation of the endobags, and sac enlargement allowing a type Ia endoleak 25 months after the procedure. He was managed with Nellix in Nellix application with concurrent visceral stent insertion (renal stent) and proximal embolisation of the aneurysm sac two years after the first treatment. The patient presented three years after the last procedure with a type Ia endoleak and progressive sac enlargement and is now scheduled for explantation.

#### Managing chimney grafting in combination with endovascular aneurysm sealing complications

Nine Ch-EVAS patients (42.9%) presented with complications which are listed in Table 3. Five patients presented with type Ia endoleak, five patients with a type Ib endoleak, and three patients with stent complications (one type III and two stent occlusions). Type Ia endoleaks were managed by Nellix stent prolongation and renal extension, two multibranched thoraco-abdominal devices, and two device explantations.

Two patients needed a branched thoraco-abdominal device for type Ia endoleak. One patient presented with type Ia endoleak six months after the first procedure (EVAS and two chimneys). Pre-operative CTA showed caudal migration of the right Nellix stent. He underwent main Nellix stent prolongation and renal stent extensions. Twenty four months after the procedure, he presented with right renal stent stenosis and underwent an endovascular angioplasty. Thirty six months after the first procedure, he presented with a large type Ia endoleak associated with sac enlargement. CTA showed that both Nellix stents had migrated caudally. He underwent an endovascular repair with an extra design branched thoraco-abdominal endoprosthesis (JOTEC EXTRADESIGN, Artivion, Kennesaw, GA, USA) associated with covered stenting of the superior mesenteric artery, occlusion of the coeliac trunk with an Amplatzer plug, and bilateral limb extensions.

The other patient with three chimneys (superior mesenteric artery and both renal arteries) presented 67 months after the first procedure, with a massive type Ia endoleak and sac enlargement (9 cm diameter) due to a dislocation of the Nellix stents. The patient underwent thoraco-abdominal multibranched device (JOTEC EXTRADESIGN, Artivion, Kennesaw, GA, USA), aorto-uni-iliac endoprosthesis, left limb embolisation, and femorofemoral bypass.^21^ He was discharged 10 days later.

One patient presented 62 months after the first procedure with rupture of a juxtarenal aortic aneurysm initially treated with Ch-EVAS (two chimneys in the renal arteries). He was known to have been treated with an ilio-mesenteric bypass after failed renal angioplasty for stent occlusion four months after the first operation. He underwent Nellix explantation with supracoeliac clamp and aortobifemoral bypass. He needed a femoral approach to suture the artery, had colic ischaemia, and underwent total colectomy and bowel resection on day one. He required haemodialysis and developed multi-organ failure and died on day three.

One patient presented first with sac enlargement with no stent migration and he benefited from two embolisations for a type Ia and type II endoleak. He presented again with sac enlargement and a persistent type Ia endoleak 24 months after the procedure and underwent Nellix explantation with aorta to the left external and right common iliac arteries with an intraperitoneal approach and infrarenal clamp. He was discharged home 11 days after the surgery after a paralytic ileus was treated medically.

Type Ib endoleaks were managed with limb extension.

Stent re-stenosis and or occlusion occurred in seven patients, but only three of them underwent a further procedure. Six patients had initial CH-EVAS and one the Nellix in Nellix adjuvant treatment. Four of them were discovered on the routine follow up CTA and did not undergo further procedures. Two of them underwent angioplasty, and one iliorenal bypass after a failed angioplasty attempt.

## Discussion

In this study, the median time of graft failure and re-interventions occurred at around three years, suggesting that patients should have had a close follow up. Graft failures were mainly stent migration with sac expansion leading to rupture, therefore patients needed re-interventions. Patients with Ch-EVAS experienced more complications than EVAS patients. All patients, even patients unfit for open surgery, need to be referred to a highly specialised centre to find the appropriate management.^22^

Although this study includes a small number of patients, the data corroborated other studies from the UK.^15^^,^^16^ Both studies showed at least a 30% of graft failure and occurred within a two year follow up. EVAS had the potential to overcome two issues: the first being prophylaxis against type II endoleaks, and the second preventing type I endoleak in the juxtarenal AAA managed by two covered stents within the endobags, allowing one to treat adverse aortic neck.^23^ Ch-EVAS was an easy adjunct to treat juxtarenal AAA, allowing the higher placement of stents, and sac filling playing the role of seal. The polymer sealing technology was reported to be able to create a seal in short, conical, and angulated necks that were high risk for EVAR.^24^ However, with the disintegration of the polymer within the bag, gutters and type I endoleak appeared. The possibility to overcome those two obstacles made this technique very promising. EVAS failure has been demonstrated, and Ch-EVAS does not seem to reduce complications either.

Concerning the management of type I endoleak, which has been the most challenging complication of the EVAS system, it was thought that open surgery with device explantation was the best option, although endovascular options have also been described to treat unfit patients. The endovascular approach relies on the extension of the aortic main body associated with chimneys or extension of pre-existent chimneys. With EVAS, patients need a very close follow up, because stent migration not sac enlargement is the main cause of device failure.

Most EVAS procedures were performed outside the IFU. Nonetheless, research indicated that adhering to the updated IFU in 2016 did not alter the results.^16^ This indicates that the observed rate of treatment failure in this study is not solely attributable to the complexity of anatomy in the aneurysms treated.

In order to fully comprehend EVAS failures, EVAS explants should be sent for further analysis, ideally with the pre-operative CTA. EVAS devices need to be analysed with the same thoroughness in vivo and ex vivo to explain the exact mechanisms of the failure. Even if the device is not commercially available, failure mechanisms need to be understood and this may help the development of future devices.^25^

EVAS devices have now been withdrawn from the market. The efficacy of combining sac filling with a stent graft designed to resist migration, such as one with active fixation, in enhancing long term durability remains unproven. However, the concept of sac anchorage does not seem to offer a lasting solution. Adjunctive devices such as the Heli-FX EndoAnchors (Medtronic Vascular, Minneapolis, MN, USA) are intended to provide fixation and sealing between the endovascular aortic graft and the native artery and have been used in conjunction with standard EVAR devices for treating short neck AAAs.^22^

The data are aligned with the surveillance algorithm proposed by Singh et al.^19^ Indeed, EVAS should not be used and patients with EVAS should be included in the surveillance programme with imaging interpreted by a designated consultant vascular interventional radiologist with expertise in EVAS, with at least a CTA every six months and explantation at a high volume specialised centre if the aneurysm sac increases more than 0.5 cm.^18^^,^^22^ The median time for graft failure and for re-interventions was respectively 47 months and 46 months in this study. All patients in whom these devices had been implanted should be enrolled early in enhanced surveillance programmes including initial CTA, clinical assessment, and subsequent duplex imaging every six months and plain abdominal radiographs to identify sac expansion, device migration, and endoleaks. If problems are identified, an additional CTA should be performed to provide more detailed assessment with discussion at subsequent multidisciplinary team meetings. Absence of endoleak does not exclude device failure. Although Nellix have been recalled, patients who have been treated with this technique need to have appropriate management and be part of an enhanced surveillance programme.

EVAS should not be used at all to treat AAA, and filling the aneurysmal sac is not a safe option. There are now a wide range of options to treat ruptured juxtarenal aortic aneurysm, including off the shelf devices, allowing patients to be treated safely, without EVAS or Ch-EVAS.

### Limitations

The retrospective and single center aspect of the study limit its interpretation. The total number of patients was relatively small, although management of complications needs to be reported to ensure patient safety. The number of patients lost to follow up is high, although they are currently being contacted. Gathering data for failed devices is mandatory to help patients treated with this system.

### Conclusion

EVAS failure rate at three years is high. An enhanced surveillance programme is essential to identify those at risk of device failure. A multidisciplinary decision in specialised centres should be documented to facilitate device failure management. CTA is suggested every six months after two years with explantation if the aneurysm sac increases more than 0.5 cm. Patients should be encouraged to undergo screening to detect graft failure and therefore undergo an elective procedure to manage EVAS complications. EVAS is not a safe option to treat AAA.

## Conflict of interest

None.

## Funding

None.

## Ethics approval

Yes.
